# Effect of PEA-OXA on neuropathic pain and functional recovery after sciatic nerve crush

**DOI:** 10.1186/s12974-018-1303-5

**Published:** 2018-09-14

**Authors:** Enrico Gugliandolo, Ramona D’amico, Marika Cordaro, Roberta Fusco, Rosalba Siracusa, Rosalia Crupi, Daniela Impellizzeri, Salvatore Cuzzocrea, Rosanna Di Paola

**Affiliations:** 1 Department of Chemical, Biological, Pharmaceutical and Environmental Sciences, University of Messina, Viale Ferdinando Stagno D’Alcontres, no 31, 98166 Messina, Italy; 2 Department of Pharmacological and Physiological Science, Saint Louis University, St. Louis, MO USA

**Keywords:** Neuroinflammation, PEA-OXA, Sciatic nerve

## Abstract

**Background:**

Animal models of sciatic nerve injury are commonly used to study neuropathic pain as well as axon regeneration. Inflammation/immune response at the site of nerve lesion is known to be an essential trigger of the pathological changes that have a critical impact on nerve repair and regeneration; moreover, the damage to peripheral nerve can cause a loss of sensory function and produces a persistent neuropathic pain. *N*-Acylethanolamines (NAEs) involve a family of lipid molecules existent in animal and plant, of which is *N-*palmitoylethanolamide (PEA) that arouses great attention owing to its anti-inflammatory, analgesic, and neuroprotective activities. The modulation of specific amidases for NAEs (and in particular NAE-hydrolyzing acid amidase NAAA, which is more selective for PEA) could be a condition to preserve its levels. Here, we investigated, in a mice model of sciatic nerve crush, the effect of 2-pentadecyl-2-oxazoline (PEA-OXA) the oxazoline of PEA that reportedly modulates activity of NAAA.

**Methods:**

In this experimental model, the mice, following the sciatic nerve crush, were treated daily with PEA-OXA at a dose of 10 mg\kg for 14 days. Therefore, we evaluated the effects of PEA-OXA on the degree of injury, on the inhibition of neuropathic pain, and on the inflammatory process, as in the improvement of reparative processes and therefore in the restoration of locomotor function.

**Results:**

Our results showed that PEA-OXA (10 mg/kg) treatment, daily, for 14 days after sciatic nerve crush, have an anti-inflammatory and neuroprotective effect and moreover have an analgesic protective effect on hypersensitivity, and improve the functional recovery after nerve crush.

**Conclusions:**

Therefore, treatment with PEA-OXA as a whole has shown a protective effect, which makes it a powerful candidate for the treatment of peripheral nerve injury and neuropathic pain.

## Background

Injury to peripheral nerve is often due to compression and cutting and through a variety of trauma, or ischemic and metabolic disorders. The damage to peripheral nerve can cause a loss of sensory function and produces a persistent neuropathic pain; injury to nerves initiates a cascade of events, which includes the degeneration of the distal part of the nerve, the increase in infiltration of inflammatory cells such as macrophages, and events that are part of a complex mechanism defined as Wallerian degeneration [1]. The immune response and the consequent inflammatory process play a key role in the pathogenesis of chronic pain, and in fact, one of the five cardinal signs of inflammation is pain, which under certain conditions, can become persistent and become chronic, even after the injury can be healed. Persistence of neuropathic pain reduces the quality of life for the patient and can lead to the failure of current therapeutic strategies [2]. In fact, a goal of modern medicine is to find an effective treatment for the management of chronic neuropathic pain. More and more interest is directed towards mediators who are able to resolve the inflammatory process, in particular different lipid mediators have been reached able to “extinguish the inflammatory process”, and then start the inflammation resolution processes [3]. In this group of substances belong *N*-arachidonoylethanolamine (an endocannabinoid) and its congeners *N*-stearoylethanolamine, *N*-oleoylethanolamine, and *N*-palmitoylethanolamine (PEA or palmitoylethanolamide) [4]. Recently, several studies have highlighted how the administration of exogenous PEA is a good pharmacological strategy against inflammatory and neurodegenerative processes [5, 6]; moreover, the pea has been shown that it possesses analgesic properties in different models of neuropathic pain [7, 8], which increases its interest as a new therapeutic approach, also considering its good safety profile [9, 10]. Fatty acid ethanolamides are degraded by different enzymes and in particular by the *N*-acylethanolamine acid amidase (NAAA) and fatty acid amide hydrolase (FAAH). And it has been seen how the inhibition of these enzymes increase the levels of PEA and therefore an inhibition of pain in sciatic pain model [11, 12]. However, the pharmacological blockage of these enzymes can lead to disorders from the metabolic point of view, since the substrates such as PEA are produced on demand, so it has been seen that from the point of view of the pharmacological approach it is more useful to be modular and not blocking the activity of these enzymes, this can be achieved with the oxazoline, PEA 2-pentadecyl-2-oxazoline of palmitoylethanolamide (PEA-OXA) [6]. PEA-OXA has been identified in several natural compounds such as coffee, and it has been observed that its effectiveness in reducing inflammation and hyperalgesia is considerably significantly greater compared to PEA [13]. Therefore, in this experimental model of sciatic nerve crush, we wanted to evaluate the combination of the protective effects of the PEA with the modulation of NAAH, in the modulation of the inflammatory process, perception of the induced pain and consequently in the repair process after the damage, through the use of PEA-OXA.

## Methods

### Animal

CD1 mice (male 25–30 g; Envigo, Milan, Italy) were placed in a controlled location (room kept at 22 ± 1 °C with 12-h dark/light cycles) and provided with standard rodent water and chow ad libitum. The animals were familiarized to their setting for 1 week. The University of Messina Review Board for the care of animals approved the research. All animal experiments observe the regulations in Italy (D.M. 116192) as well as the EU regulations (O.J. of E.C. L 358/1 12/18/1986).

### Surgery

For nerve crush, it was conducted with minor changes to what was seen previously [14], mice were anesthetized with 2% isofluorane/oxygen, lateral thigh shaved, and a 1 cm incision in the skin made over the lateral femur. The muscle layers were split with blunt scissors, the sciatic nerve localized and crushed with an ultra-fine, smooth, straight hemostat (tip width 0.6 mm/ Fine Science Tools) for 30 s [14].

### Experimental groups

Mice were randomly divided into the following groups (*n* = 10 for each group).

PEA-OXA was obtained according to the synthesis described previously by [13].

Vehicle: mice were subjected to sciatic nerve crush as described above, and vehicle (carboxymethylcellulose (CMC) 2.5% p/p in water) was orally administered for 14 days.

PEA\OXA: mice were subjected to sciatic nerve crush as described above, and PEA-OXA (10 mg/kg) (dissolved in carboxymethylcellulose (CMC) 2.5% p/p in water) was orally administered daily for 14 days.

SHAM: mice were subjected to the surgical procedures as above group except that sciatic nerve crush was not applied, and vehicle was administered at 1 day after.

Sham + PEA-OXA: mice were subjected to the surgical procedures as above group except that sciatic nerve crush was not applied, and PEA-OXA (10 mg/kg) (dissolved in carboxymethylcellulose (CMC) 2.5% p/p in water) was orally administered daily for 14 days.

As described below, mice (*N* = 10 from each group for each parameter; calculated using the statistical test a priori power analyzes of the G-power software) were sacrificed 14 days after sciatic nerve crush.

### Histological analysis

At the end of the 14 days, for histological examination standard hematoxylin and eosin (H&E) staining was performed as seen previously [15], sciatic nerve tissue specimens were then observed under an optical microscope (Leica QWin V3, Cambridge, UK). sciatic nerves, scores of 0, 1, 2, 3, and 4 indicate 0, 1–25, 26–50, 51–75, and .75% infiltration, respectively [16]. Identification of mast cells was performed in sciatic nerve tissue specimens sections by blue toluidine staining as described previously [15].

### Western blot analysis

Western blot analysis were performed on the lumbar portion of the spinal cord; the samples were homogenized in lysis buffer. Cytosolic and nuclear extracts were prepared as described previously [15]. The filters were probed with specific Abs: anti-NF-κB p-65 (1:1000; Santa Cruz Biotechnology), IκB-α (1:1000; Santa Cruz Biotechnology), anti-TNF-α (1:1000; Santa Cruz Biotechnology), anti-IL-1β (1:1000; Santa Cruz Biotechnology), anti-Bax (1:500; Santa Cruz Biotechnology), anti-Bcl-2 (1:500; Santa Cruz Biotechnology), anti-caspase-3 (1:500; Santa Cruz Biotechnology), anti-c-fos (1:500; Santa Cruz Biotechnology), anti-nerve growth factor (NGF) (1:1000; Santa Cruz Biotechnology), anti-NAAA (1:500 Sigma–Aldrich Corp), or anti β-III-tubulin (1:1000 cell signaling) in 1 × PBS, 5% *w*/*v* nonfat dried milk, 0.1% Tween-20 at 4 °C, overnight. To ascertain that blots were loaded with equal amounts of proteins, they were also incubated in the presence of the antibody against β-actin protein (cytosolic fraction 1:500; Santa Cruz Biotechnology) or lamin A/C (nuclear fraction 1:500 Sigma–Aldrich Corp.). Signals were detected with enhanced chemiluminescence (ECL) detection system reagent according to the manufacturer’s instructions (Thermo, USA). The relative expression of the protein bands was quantified by densitometry with BIORAD ChemiDocTM XRS+software and standardized to β-actin and lamin A/C levels. Images of blot signals (8 bit/600 dpi resolution) were imported to analysis software (Image Quant TL, v2003). The blot was stripped with glycine 2% and reprobed several times to optimize detection of proteins and to visualize other proteins without the need for multiple gels and transfers.

### Immunofluorescence

After deparaffinization and rehydration, detection of β-III-tubulin was carried out after boiling the tissue sections in 0.1 M citrate buffer for 1 min as described previously [17]. Non-specific adsorption was minimized by incubating in 2% (vol/vol) normal goat serum in PBS for 20 min. Sections were incubated with β-III-tubulin primary antibodies (1:400 cell signaling) in a humidified chamber overnight at 37 °C. Sections were then incubated with secondary antibody: fluorescein isothiocyanate-conjugated anti-mouse Alexa Fluor-488 (1:2000, Molecular Probes, Monza, Italy) or Texas Red-conjugated anti-rabbit Alexa Fluor-594 (1:1000, Molecular Probes) for 1 h at 37 °C. For nuclear staining, 2 μg/ml 4′,6′-diamidino-2-phenylindole (DAPI; Hoechst, Frankfurt, Germany) in PBS was added. Sections were observed using a Leica DM2000 microscope (Leica, Milan, Italy). Optical sections of fluorescence specimens were obtained using a HeNe laser (543 nm), an ultraviolet laser (361–365 nm), and an argon laser (458 nm) at a one-min, 2-s scanning speed with up to eight averages; 1.5-μm sections were obtained using a pinhole of 250. The same settings were used for all images obtained from the other samples that had been processed in parallel. Digital images were cropped, and figure montages prepared using Adobe Photoshop 7.0 (Adobe Systems; Palo Alto, CA, USA). The co-localization of images was examined with ImageJ software (National Institutes of Health) as described previously [18].

### Tunel staining

TUNEL staining protocol was according to a Roche protocol. Paraffin-embedded sections were dewaxed in xylene and rehydrated in a graded ethanol series to water, permeabilized with citrate buffer 0.1 M, and then incubated in TUNEL reaction mixture for 60 min at 37 °C in the dark. The tissue was then rinsed in PBS three times for 5 min and then observed using an excitation wavelength in the range of 520–560 nm (maximum 540; green) and in the range of 570–620 nm (maximum 580 nm; red).

### Behavioral testing

All the behavioral testing was performed in a blinded fashion.

### Thermal hyperalgesia (paw withdrawal test)

To assess hind paw heat sensitivity, Hargreaves’ test was conducted using a plantar test device (plantar test; Ugo Basile, Italy) [19] as seen previously [20]. Animals were allowed to freely move within an open-topped transparent plastic box on a glass floor 20 min before the test. A mobile radiant heat source was then placed under the glass floor and focused onto the hind paw. Paw withdrawal latencies were measured with a cutoff time of 15 s to prevent tissue damage. The heat stimulation was repeated three times with a 10-min interval to obtain the mean latency of paw withdrawal. Results are expressed as paw withdrawal latency(s).

### Mechanical allodynia dynamic aesthesiometer

Mechanical allodynia was evaluated using the Dynamic Plantar Aesthesiometer (Ugo Basile). This equipment employs a single non-flexible filament (0.5 mm diameter) to apply an increasing force to the plantar surface of the mouse hind paw. Animals were placed in a cage with a wire mesh floor and allowed to acclimatize before testing. The filament was applied to the plantar area of the hind paw and it began to exert an increasing upward force, reaching a maximum of 30 g in 10 s, until the paw was withdrawn. The withdrawal threshold was defined as the force, in grams, at which the mouse withdrew its paw. Withdrawal was determined three times, and the reported value is the mean of the three evaluations.

### Beam walking

Coordination and balance were assessed, with minor changes to what has been seen previously [21–23], by measuring the ability of mice to traverse a wooden beam (1 m × 26 mm), in order to reach a dark, enclosed safety platform containing food and bedding. Mice were trained over two consecutive days (three trials per day) by placing them at the starting point and allowing them to traverse a 70-cm section of the beam. Once trained, mice were tested using three consecutive trials. Observers were blinded to the study, and a video camera was used to record three trials. The time taken for the mouse to cross the full length of the beam to the goal box was recorded for a maximum time of 120 s. If the mouse fell before reaching the goal box, the time was recorded as 120 s. The time spent in a frozen posture and number of right paw slips were also noted. Data from three trials were averaged for each, and the difference between the experimental groups were analyzed by two-way ANOVA followed by Fisher PLSD post hoc tests.

### Functional studies

The sciatic functional index (SFI) was evaluated, as previously described [24], 7 and 14 days after sciatic nerve crush. The paw prints were recorded by moistening the back legs of each animal with blue ink and making them walk without assistance along a 6 × 44 cm corridor, on white sheets of paper. The animals’ paw prints were recorded, and two measurements were taken: (i) the print length (PL), corresponding to the distance from the heel to the third toe, and (ii) the toe spread (TS), corresponding to the distance from the first to fifth toe. Both measurements were taken from injured (E, for experimental) as well as non-injured (N, for normal) sides, and the SFI was calculated according to the formula:

$$ \mathrm{SFI}=118.9\left(\frac{\mathrm{ETS}-\mathrm{NTS}}{\mathrm{NTS}}\right)-51.2\left(\frac{\mathrm{EPL}-\mathrm{NPL}}{\mathrm{NPL}}\right)-7.5 $$

The SFI value varies from 0 to − 100, with 0 corresponding to normal function and − 100 corresponding to complete dysfunction.

### Materials

PEA-OXA was kindly supplied by Epitech Group SpA (Saccolongo, Italy). Unless otherwise stated, all compounds were obtained from Sigma–Aldrich. All solutions used for in vivo infusions were prepared using non-pyrogenic saline (0.9% NaCl; Baxter Healthcare Ltd., Thetford, Norfolk, UK).

### Statistical evaluation

All values are expressed as mean ± standard error of the mean (S.E.M.) of *N* observations. For in vivo studies, *N* represents the number of mice used. In experiments involving histology, immunohistochemistry, and immunofluorescence, the figures shown are representative of at least three experiments performed on different days on tissue sections collected from all animals in each group. The results were analyzed by one-way ANOVA followed by a Bonferroni post hoc test for multiple comparisons. A *P* value of less than 0.05 was considered significant.

## Results

### Effect of PEA-OXA on histological changes after sciatic nerve crush

**Fig. 1:**
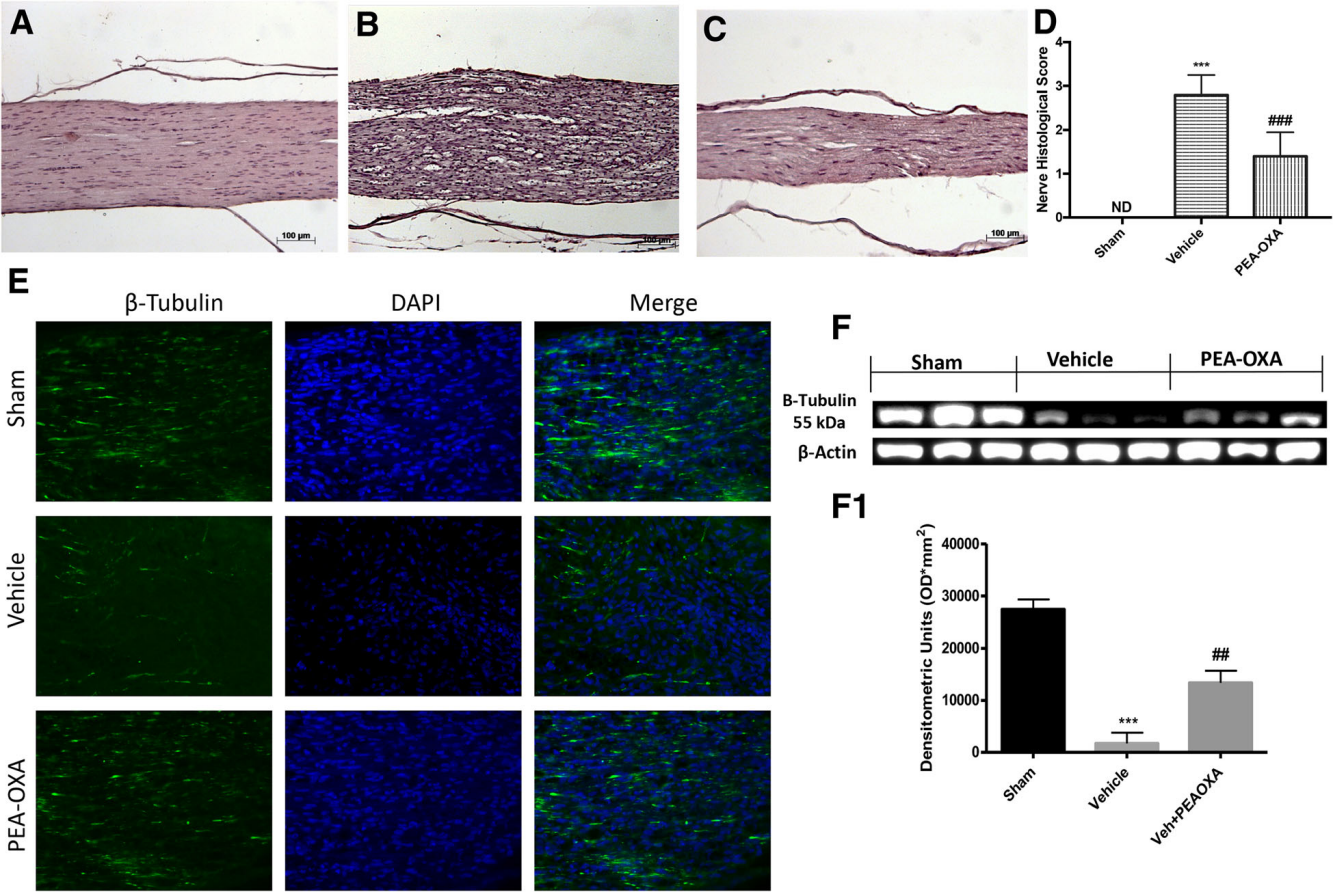
Effect of PEA-OXA on histological changes and mast cell density in sciatic nerve. **a** Sciatic nerve structure from sham mice. **b** Nerve from the vehicle group exhibited, 14 days after sciatic nerve crush, the several areas of edema with an abundant presence of infiltrate and degraded myelin sheets. **c** PEA-OXA treatment significantly reduces the presence of edema and infiltrate, as indicated by nerve histological score in **d**. **e** Immunofluorescent staining for β-III-tubulin on longitudinal sections of the sciatic nerve. Following crushing of the sciatic nerve and treatment with PEA-OXA, there is a significant increase in expression of β-III-tubulin, compared to the vehicle group. **f**, **f1** Western blot analysis of the lumbar portion of the spinal cord showed the expression and relative densitometric analysis for β-III-tubulin. The PEA-OXA treatment was able to significantly increase the β-III-tubulin compared with vehicle group. Each data are expressed as mean ± SEM from *N*  =  10 mice for each group. ***p < 0.001 vs corresponding sham group. ##*p* < 0.01 vs vehicle group. ###*p* < 0.001 vs. vehicle group

Sciatic nerve from the sham group showed a normal structure of sciatic nerve (Fig. 1a), and 14 days after sciatic nerve crush, the sciatic nerve from the vehicle group exhibited several areas of edema with an abundant presence of infiltrate and degraded myelin sheets as show in Fig. 1b, PEA-OXA treatment significantly reduce the presence of edema and infiltrate (Fig. 1c) compared with the vehicle group as shown by the nerve histological score Fig. 1d. After, as a marker of an efficient nerve repair process, we evaluated the expression of β-III-tubulin, both in the spinal cord and in the injured nerve. The immunofluorescent staining β-III-tubulin on longitudinal sections of the sciatic nerve shows that following crushing of the sciatic nerve and treatment with PEA-OXA there is a significant increase in expression of β-III-tubulin as shown in (Fig. 1e), compared to the vehicle group (Fig. 1e). Also, western blot analysis on the lumbar portion of the spinal cord showed a significant reduction in β-III-tubulin expression 14 days after sciatic nerve crush, whereas PEA-OXA treatment daily for 14 days after sciatic nerve crush significantly increased β-III-tubulin expression compared to the vehicle group, as shown in Fig. 1f, f1.

### Effect of PEA-OXA on mast cells density and pain behavior

**Fig. 2:**
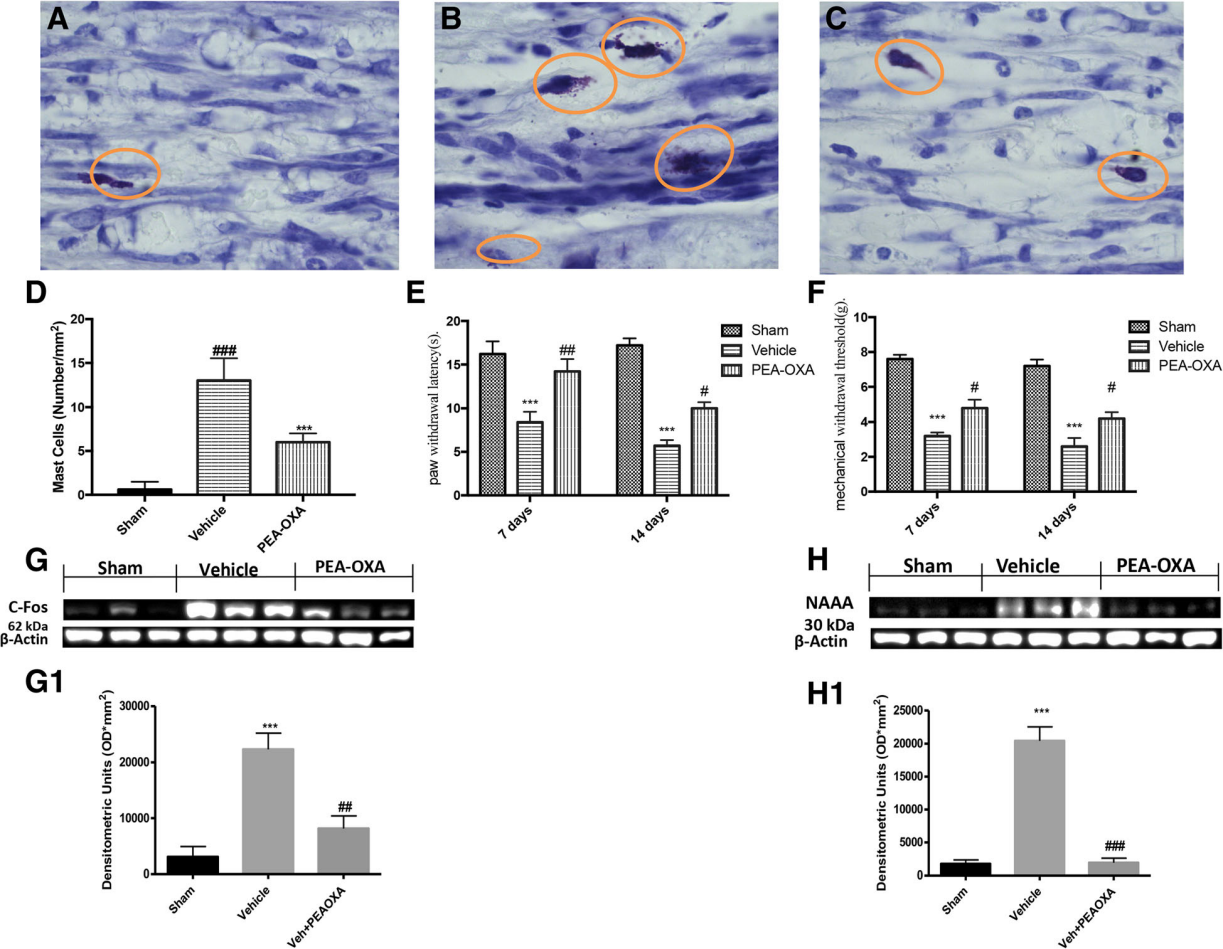
Effect of PEA-OXA on mast cells density and pain behavior. The blue toluidine staining for highlight the mast cells, **a** for sham group. **b** 14 days after sciatic nerve crush, there is a significant increase in mast cell number in sciatic nerve from vehicle group. **c** How daily treatment with PEA-OXA significantly reduces the presence of mast cell compared to vehicle group as shown by **d**. **e**, **f** The analgesic effect of daily treatment of PEA-OXA 10 mg\kg, on thermal hyperalgesia (Plantar test) and mechanical allodynia. **g**, **g1** The c-fos expressions by western blot analysis in the lumbar portion of the spinal cord. **h**, **h1** Western blot analysis on the lumbar portion of the spinal cord for NAAA expressions. Each data are expressed as mean ± SEM from *N*  =  10 mice for each group. ***p < 0.001 vs corresponding sham group. #*p* < 0.05 vs. vehicle group, ##*p* < 0.01 vs vehicle group, ###*p* < 0.001 vs. vehicle group

We subsequently assessed whether treatment with PEA-OXA had effects on the number of mast cells that play a key role in the inflammatory process and in particular in the development of hyperalgesia. As shown in Fig. 2b, 14 days after sciatic nerve crush, there is a significant increase in mast cell number, compared with uninjured nerve from the sham group Fig. 2a, d. Daily treatment with PEA-OXA significantly reduced the presence of mast cell compared to the vehicle group as shown by Fig. 2c, d. As shown in Fig. 2e, mice from the vehicle group present a significant increase in the response to thermal stimuli, from days 7 to 14 after nerve crush when compared with basal response, and as also shown in Fig. 2e, mice treated with PEA-OXA show a significant reduction of their pain threshold compared to the vehicle group. Figure 2f shows, the sciatic nerve crush produced a reduction in the nociceptive threshold to mechanical stimuli from days 7 to 14 in the operated paw. Instead, daily treatment with PEA-OXA at a dose of 10 mg\kg significantly increased the thresholds to mechanical stimuli. Therefore, as a marker of the activation of neurons by algesic stimuli, we evaluated the expression of c-fos, and western blot analysis on the lumbar portion of the spinal cord showed that 14 days after the sciatic nerve crush there is a significant increase in c-fos expression in mice from the vehicle group. Instead, treatment with PEA-OXA was able to significantly reduce the c-fos expression as shown in Fig. 2g, g1. As a mechanism of action of PEA-OXA has been proposed, the combination of the beneficial effects of PEA with those of the inhibition of NAAA, the principal enzyme is responsible for the degradation of PEA [25]. Therefore, we evaluated the expression of NAAA at the spinal level, as shown in Fig. 2h, h1. Fourteen days after the crush to sciatic nerve, there was a significant increase in the expression of NAAA whereas daily treatment with PEA-OXA at the dose of 10 mg\kg significantly reduced the expression of NAAA induced by the sciatic nerve crush.

**Fig. 3:**
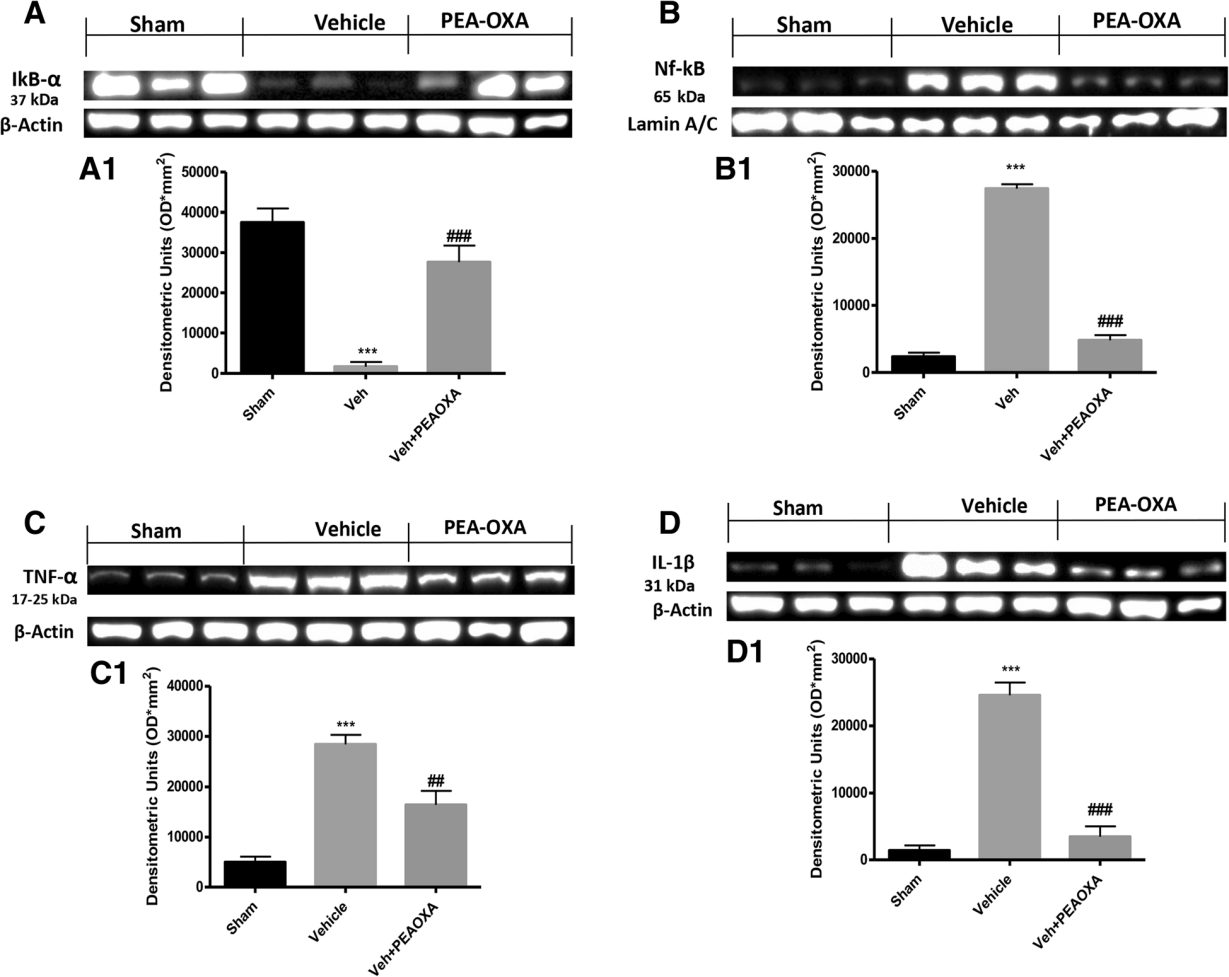
Effect of PEA-OXA on inflammatory response after sciatic nerve crush. Western blot analysis of the lumbar portion of the spinal cord showed the expression and relative densitometric analysis for: Ikb-α (**a**, **a1**); for Nf-κb (**b**, **b1**); for TNF-α (**c**, **c1**); and for IL-1β (**d**, **d1**). Each data are expressed as mean ± SEM from *N*  =  10 mice for each group. ***p < 0.001 vs corresponding sham. ##*p* < 0.01 vs vehicle group, ###*p* < 0.001 vs. vehicle group

### Effect of PEA-OXA on inflammatory response after sciatic nerve crush

As showed by our results, crush to sciatic nerve produces an important inflammatory response, which is a key event both for the development of neuropathic pain and for neurodegenerative processes. In particular, as shown in Fig. 3a, a1, b, b1, western blot analysis of the lumbar portion of the spinal cord showed that, 14 days after sciatic nerve crush, in the vehicle group, there is a significant increase in Ikb-α degradation and consequently an increase in Nf-κb translocation to nucleus, this is a key event both for the inflammatory response and for changes in the transcription of different genes. Compared to the vehicle group, PEA-OXA treatment significantly reduced the Ikb-α degradation and Nf-κb translocation (Fig. 3a, a1. b, b1). Moreover, as shown in Fig. 3c, c1, d, d1, crush to sciatic nerve produce a significant increase in proinflammatory cytokines TNF-α and IL-1β, whereas daily treatment with PEA-OXA for 14 days after sciatic nerve crush significantly reduced the expression of TNF-α and IL-1β.

### Effect of PEA-OXA on astrocytes and microglia activation after sciatic nerve crush

**Fig. 4:**
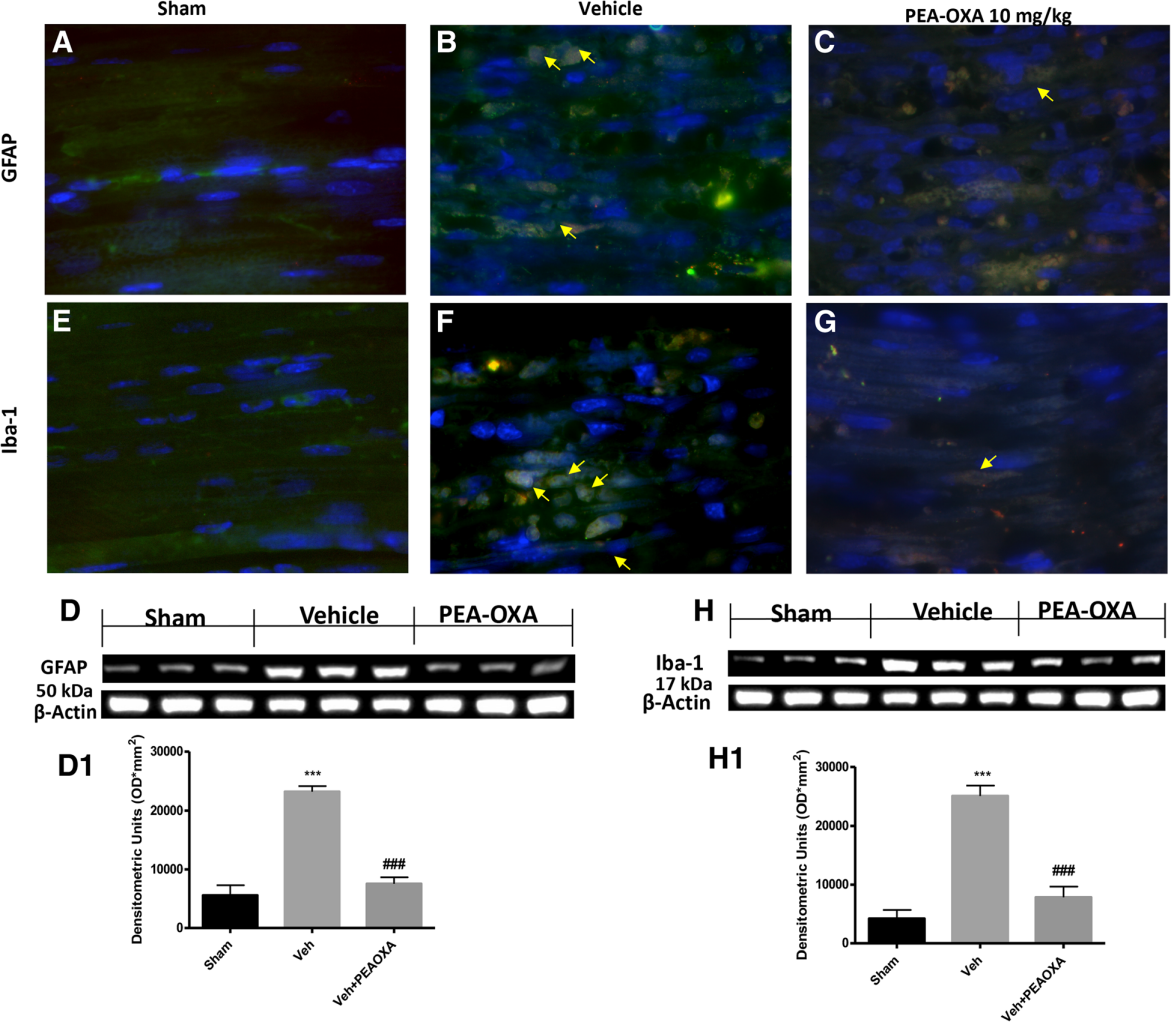
Effect of PEA-OXA on astrocytes and microglia activation after sciatic nerve crush. The immunofluorescent staining on longitudinal sections of the sciatic nerve for GFAP and Iba-1, showed a significant increase in GFAP and Iba-1 expression in the vehicle group (**b**, **f**, respectively) compared to sham mice (**a**, **e**, respectively). PEA-OXA treatment significantly reduces the expression for GFAP and Iba-1 compared to the vehicle group, **c**, **g**, respectively. The western blot analysis of the lumbar portion of the spinal cord showed the expression and relative densitometric analysis for GFAP and Iba-1 (**d**, **d** and **h**, **h1,** respectively). Each data are expressed as mean ± SEM from *N*  =  10 mice for each group. ***p < 0.001 vs corresponding sham. ###*p* < 0.001 vs. vehicle group

Astrocytes and microglia activation play a critical role in neuroinflammation and in neuropathic pain state. When compared to the sham group (Fig. 4 panel a, for glial fibrillary acidic protein (GFAP) and panel e for Iba-1, see yellow arrows), immunofluorescence evaluation of GFAP and ionized calcium binding adaptor molecule 1 (Iba1) revealed a significant increase in GFAP and Iba-1-positive cell as shown in Fig. 4b, f, respectively. Treatment with PEA-OXA at a dose of 10 mg\kg post crush to sciatic nerve significantly reduces the number of positive cells for GFAP and Iba-1 Fig. 4c, g, respectively, compared to the vehicle group. As shown by western blot analysis Fig. 4d, d1, h, h1 respectively, also at the spinal cord level, we found an increase in GFAP and Iba-1, 14 days after sciatic nerve crush. Instead, daily treatment with PEA-OXA significantly reduces the GFAP and Iba-1 expression, (Fig. 4d, d1, h, h1 respectively).

### Effect of PEA-OXA on apoptotic pathway after sciatic nerve crush

**Fig. 5:**
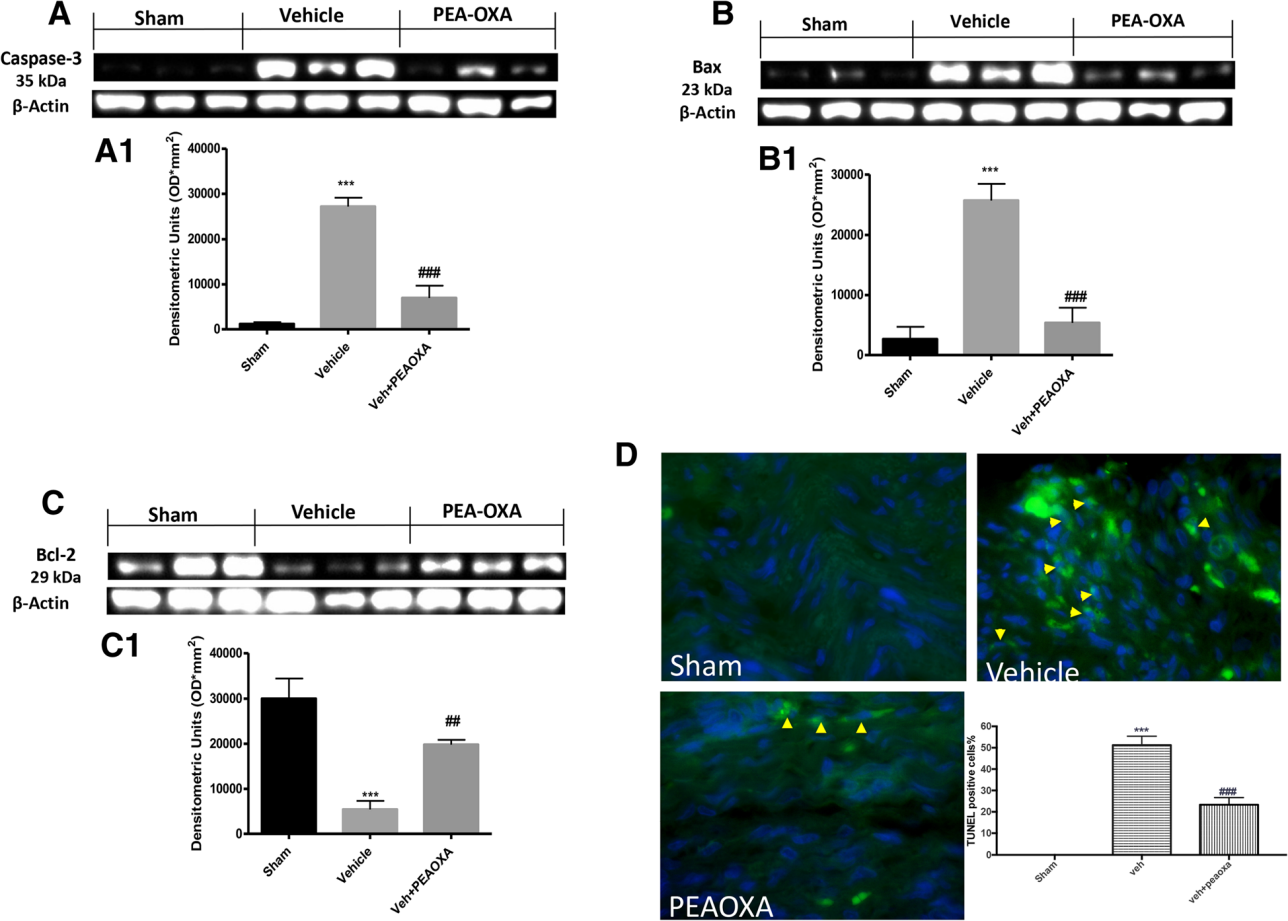
Effect of PEA-OXA on apoptotic pathway after sciatic nerve crush. Western blot analysis of the lumbar portion of the spinal cord showed the expression and relative densitometric analysis for caspase-3 (**a**, **a1**); Bax (**b**, **b1**); Bcl-2 (**c**, **c1**). **d** The TUNEL staining on longitudinal section of sciatic nerve tissue. Each data are expressed as mean ± SEM from *N*  =  10 mice for each group. ***p < 0.001 vs corresponding sham group. ##*p* < 0.01 vs vehicle group. ###*p* < 0.001 vs. vehicle group

Western blot analysis on the lumbar portion of the spinal cord showed an increase in activation of the apoptotic process. In particular, 14 days after sciatic nerve crush, in mice from the vehicle group, there is a significant increase in expression for the pro-apoptotic protease caspase-3, whereas the treatment with PEA-OXA significantly reduces the expression of caspase-3, as shown in Fig. 5a, a1. Crush of sciatic nerve also produces an alteration on expression of a pro-apoptotic protein Bax and Bcl-2, an antiapoptotic protein as shown in Fig. 5b, b1 and c, c1, respectively. In fact, compared with sham mice, in the vehicle group, there is a significant increase in Bax expression and consequent reduction in Bcl-2 expression, whereas PEA-OXA treatment significantly reduces the expression of BAX and increases the Bcl-2 expression (Fig. 5b, b1, c, c1, respectively). Moreover, TUNEL assay is used to identify cells undergoing apoptosis in sciatic nerve tissue. As shown in Fig. 5d, the vehicle group showed a significant increase in number of cells undergoing apoptosis as indicated by intense staining, compared with the sham group. Treatment with PEA-OXA (10 mg\kg) significantly decreases the apoptotic process in sciatic nerve tissue.

### Effect of PEA-OXA on NGF expression and functional recovery after sciatic nerve crush

**Fig. 6:**
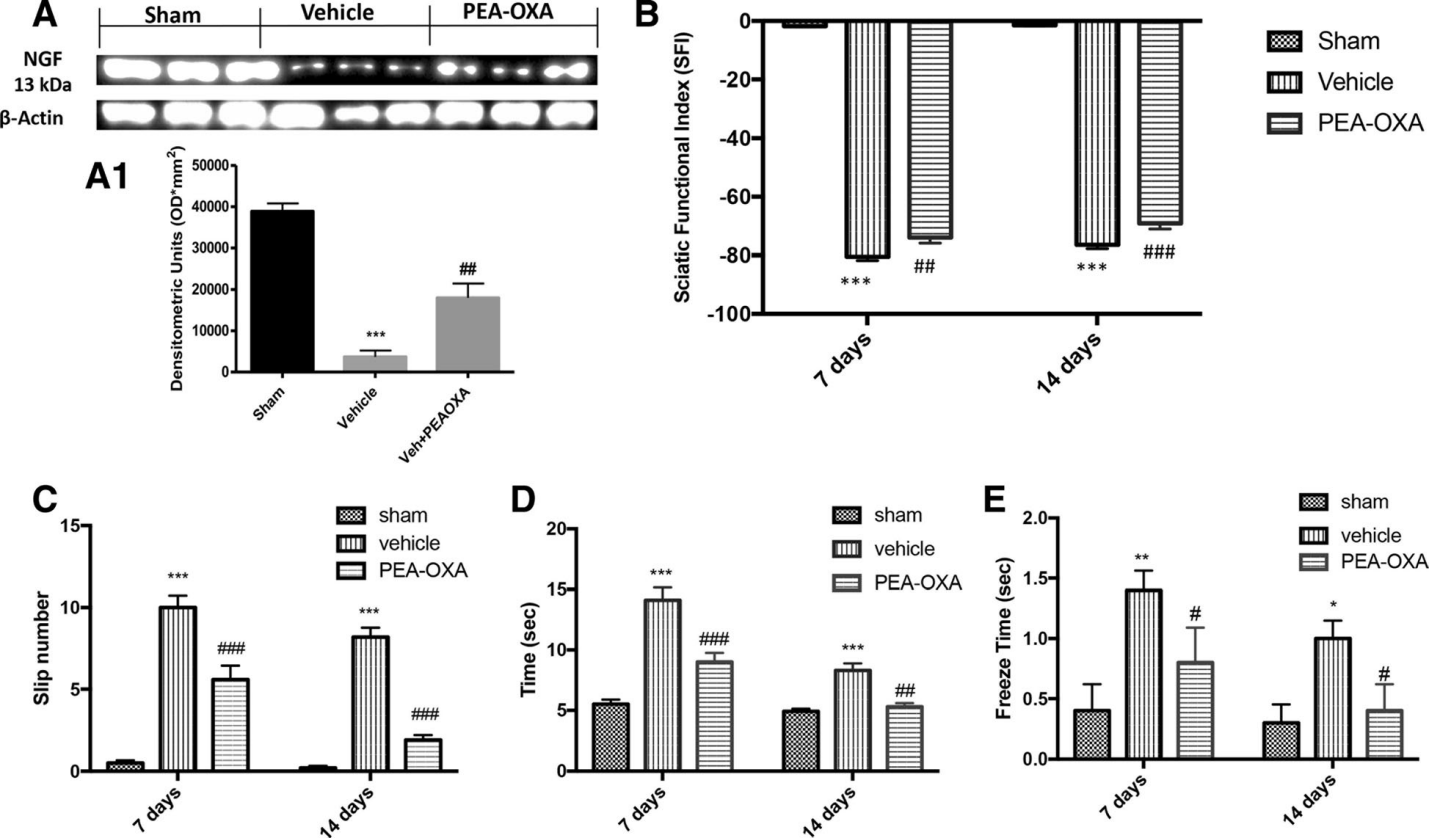
Effect of PEA-OXA on neural regeneration and functional recovery after sciatic nerve crush. **a** The western blot analysis of the lumbar portion of the spinal cord showed the expression and relative densitometric analysis for NGF; the PEA-OXA treatment was able to significantly increase the NGF compared with vehicle group. As an index of functional nerve recovery, we evaluated the sciatic functional index SFI (**b**), the SFI value varies from 0 to − 100, with 0 corresponding to normal function and − 100 corresponding to complete dysfunction. For beam walk test to assess motor coordination improvement after sciatic nerve crush, the test consists of evaluating the ability of mice to cross a wooden beam, the PEA-OXA treatment significantly improves the motor coordination when compared with the vehicle group. In fact, the mice of the vehicle group showed a greater number of sliding of the paw (**c**), a greater time of crossing of the beam (**d**), and a greater freeze time spent during the crossing (**e**). Each data are expressed as mean ± SEM from *N*  =  10 mice for each group. ***p < 0.001 vs corresponding sham group. ##*p* < 0.01 vs. vehicle group. ###*p* < 0.001 vs. vehicle group

Subsequently, we evaluated the effects of treatment with PEA-OXA on the improvement of reparative processes. Therefore, we evaluated the expression at the spinal level of the neurotrophic factor NGF, which plays a key role in neuronal survival. Western blot analysis showed that following the sciatic nerve crush in the vehicle group there is a significant reduction in the expression of NGF whereas the treatment with PEA-OXA had a protective effect in increasing the expression of NGF compared to the vehicle group as shown in Fig. 6a, a1. Subsequently, we evaluated the sciatic functional index SFI, which is an index of a functional nerve recovery. As shown in Fig. 6b, the mice from the vehicle group show a significant increase in SFI value, whereas the treatment with PEA-OXA was able to improve a functional recovery as indicated by a reduction in SFI compared to the vehicle group. We also used the beam walk test to assess motor coordination improvement after sciatic nerve crush, and the test consists of evaluating the ability of mice to cross a wooden beam, as shown in Fig. 6b, the mice of the vehicle group used a longer time to cross the beam after crushing the sciatic nerve, compared to the PEA-OXA group. Moreover, the treatment with PEA-OXA for 14 days reduced the number of paw slips and freezing time (Fig. 6a, c, respectively) during the crossing of the beam respect to the vehicle group, indicating a better recovery in the motor coordination for the PEA-OXA group following the crushing of the sciatic nerve.

## Discussion

Damage to peripheral nerves often produces a condition of neuropathic pain, characterized by an increase in painful sensitivity, such as hyperalgesia and allodynia. Furthermore, damage to the nerves produces immunological and neuronal changes and also in the expression of genes and proteins even at the level of the spinal cord [26]. Moreover, the compression of the peripheral nerves is often also associated with the loss of motor function, mainly due to an insufficient regeneration of the nerve. The therapeutic approach to this type of pathology and in particular to neuropathic pain, is a clinical problem [27]. In this study, we used a sciatic nerve crush model, one of the most used models for studying cellular and molecular mechanisms in the peripheral nerve [28], to study the effects of PEA-OXA on pain inhibition and pathological processes after crush to the sciatic nerve. PEA-OXA is the oxazoline (2-pentadecyl-2-oxazoline) of palmitoylethanolamide (PEA), a fatty acid amide belonging to the family of *N*-acylethanolamines (NAEs). Numerous studies show that PEA is an important endogenous mediator in controlling the inflammatory and analgesic phenomena [8, 29] with neuroprotective effect [6]. In particular, a recent clinical study has highlighted the role of PEA as a valid therapeutic strategy for the treatment of nerve compression pain: such as the treatment of sciatic nerve pain and carpal tunnel syndrome; in addition, clinical trials have shown that no adverse effects are reported for PEA, making PEA a valid and safe alternative to opioids and co-analgesics in the treatment of neuropathic pain [9]. Recently, it has been seen that the pharmacological inhibition of NAAA produces a marked analgesic and anti-inflammatory effect [30]; therefore, treatment with PEA-OXA combines the known effects of PEA with those of NAAA inhibition, which has among other effects, the increase of endogenous endocannabinoid levels [25]. Therefore, this particular mechanism of action enhances the already known therapeutic properties of PEA, and in fact, a recent study has shown that compared to PEA the same dose of PEA-OXA produces a significant improvement in analgesic and anti-inflammatory action [25]. So, in this study, the histological analysis of the sciatic nerve showed that 14 days after the sciatic nerve crush, there is a significant presence of edema, infiltration, and degradation of the myelin layer. Instead, compared with vehicle, the PEA-OXA treatment at dose of 10 mg\kg daily for 14 days following the sciatic nerve crush has had a beneficial effect. In fact, the histological analysis shows a significant reduction of edema and infiltration and therefore a beneficial effect in axonal regeneration. As an indicator of an efficient reparative process, we evaluated the expression of β-III-tubulin both at the spinal level and in the sciatic nerve [31, 32], our results showed a significant reduction in the expression of β-III-tubulin both at the spinal level and in the sciatic nerve, following the sciatic nerve crush. When compared to the vehicle group, daily treatment with PEA-OXA at a dose of 10 mg\kg has significantly increased the expression of β-III-tubulin both at the spinal level and the damaged sciatic nerve. Subsequently, we assessed the presence of mast cells following the damage and if the treatment with PEA-OXA was able to have a protective effect. In fact, numerous evidence suggest that mast cells play a key role in the development of the inflammatory process and in the generation of neuropathic pain following the peripheral nerve injury [33]. It has been seen that mast cells accumulate near the area of the injured nerve [34], where they are responsible for the release of various proinflammatory mediators such as TNF-α and IL-1β [35], and these factors are also responsible for the recruitment of leukocytes. In addition, the activated mast cells are responsible for the release of substances that sensitize the nociceptors and contribute to hyperalgesia among these in particular histamine [33]. In fact, blockers of histamine receptors alleviate or they inhibit neuropathic pain [36]. Our results show an increase in mast cells 14 days after sciatic nerve crush, whereas daily treatment with PEA-OXA at a dose of 10 mg\kg significantly reduced the number of mast cells in the injured nerve. In fact, we have found that the sciatic nerve crush produces a significant diminution of the nociceptive threshold to mechanical and thermal stimuli. Daily treatment for 14 days with PEA-OXA at a dose of 10 mg\kg was able to significantly increase the pain threshold to mechanical and thermal stimuli. Another way to monitor the activation of the nociceptive pathway is by measuring the levels of c-fos [37], and in fact, c-fos is recognized as a marker of activity of neurons excited by algesic stimuli and has been seen to increase chased to damage to the peripheral nerves together with the mechanic allodynia and thermal hyperalgesia [38]. In fact, at the level of the lumbar portion of the spinal cord, we found a significant increase in the expression of c-fos, 14 days after the sciatic nerve crush, while treatment with PEA-OXA significantly reduced the expression of c-fos, consistently with the decrease of the mechanical allodynia and thermal hyperalgesia that we observed. In addition, the mechanism of the analgesic effect of PEA-OXA involves the combination of the already known analgesic effects of PEA with those of the inhibition of the main enzyme responsible for its degeneration NAAA [25], it has also been seen that the inhibition of NAAA produces an analgesic effect [39]. In fact, we found at the spinal level a significant increase in the expression of NAAA following sciatic nerve crush, whereas the daily treatment for 14 days with PEA-OXA at a dose of 10 mg\kg significantly reduced the expression of NAAA compared with the vehicle group. We subsequently assessed whether sciatic nerve damage could produce changes in protein and gene expression even at the spinal level. In particular, by western blot analysis of the lumbar portion of the spinal cord, we first evaluated the involvement of the inflammatory process, nerve damage has been shown to produce an important inflammatory response, and inhibition of this process is a strategy for the treatment of neuropathic pain [40]. Our results showed that 14 days after sciatic nerve crush, at the spinal cord level of the mice from the vehicle group, there was a significant increase in Ikb-α degradation and therefore a consequent increase in Nf-κB translocation, and the activation of the Nf-κb pathway is a pivotal event in a whole series of changes, as in gene expression, and in the increase of the expression of different proteins (such as cytokines and chemokines) neurotransmitters and therefore in painful hypersensitivity [41]. Ours results showed a significant increase in Ikb-α degradation and consequent Nf-kB translocation into the nucleus, 14 days after sciatic nerve crush, whereas PEA-OXA treatment exerts a significantly protective effect; furthermore, at the lumbar spinal level, from the vehicle group, we found a significant increase in the expression of the proinflammatory cytokines TNF-α and IL-β while PEA-OXA treatment daily for 14 days following the sciatic nerve crush significantly reduces the expression of proinflammatory cytokines TNF-α and IL-1β. A typical sign of damage to the central nervous system is the increase in reactive astrocytes and of microglia as indicated by an increase in GFAP and Iba-1 expression [42, 43]. In addition, several evidence show how microglia and astrocyte are activated, at the spinal level, in the neuropathic pain state [44, 45]. Our results show that both at the damaged nerve level and at the spinal cord level, there is a significant increase in the expression of GFAP and Iba-1, in the vehicle group compared to sham mice. The treatment with PEA-OXA has significantly reduced, compared to the vehicle group, the expression of GFAP and Iba-1 both at the level of the sciatic nerve and at the spinal level. After the damage to the peripheral nerves following different events such as the inflammatory response that we have observed, the Wallerian degeneration and neuronal apoptosis, in particular the neuronal apoptosis, plays a key role as an excessive activation of this process can compromise the efficiency of the reparative processes [46]. Therefore, we evaluated the expression of caspase-3 as a marker of the process of programmed cell death (apoptosis) [47]. At the spinal level, we found that 14 days after sciatic nerve crush there was a significant increase in caspase-3 expression, whereas daily PEA-OXA treatment significantly reduced caspase-3 expression. Furthermore, to indicate an increase in the apoptotic process we found in the vehicle group an increase in the ratio between Bax and Bcl-2, two factors involved in the regulation of this process [48]; also in this case, the PEA-OXA treatment had a protective effect. Moreover, through TUNEL staining, we evaluated the apoptotic process in the sciatic nerve tissue. Fourteen days after sciatic nerve crush, the vehicle group showed a significant increase in the intensity of TUNEL staining. Instead, when compared to the vehicle, treatment with PEA\OXA 10 mg\kg significantly reduced the number of apoptotic cells. In opposition to the apoptotic process, there are the reparative processes of neuronal growth, and in these type of processes, a key role is played by the growth factors like the nerve growth factor (NGF) [49]. Therefore, consistent with what was observed for the apoptotic process, at the spinal level, we found a significant reduction in NGF expression 14 days after the sciatic nerve crush, while daily treatment with PEA-OXA significantly increased the expression of NGF when compared to the vehicle group. This is a key step in neuroprotective action of the PEA-OXA. Indeed, NGF plays a fundamental role in the function and survival of neurons in the peripheral nervous system [50]. Moreover, it has been seen that a deficit in NGF is often associated with various neurodegenerative diseases [51], and in particular, neurodegenerative processes together with neuroinflammation play a key role in the pathogenesis and presence of neuropathic pain [52]. Finally, we evaluated the events described so far from a macroscopic point of view, and in fact, we evaluated the recovery of motor functions, by evaluating the ability of mice to cross a wooden beam (beam walking test) and the sciatic functional index (SFI). The calculation of SFI is often used to evaluate Functional recovery following sciatic nerve injury [53]. The SFI is scaled such that − 100 represents a complete nerve injury and 0 represents normal function. Our results show how when compared to the vehicle, treatment with PEA-OXA significantly improves functional recovery following sciatic nerve injury from both 7 and 14 days. We observed both 7 and 14 days after the sciatic nerve crush that the mice of the vehicle group took a significantly longer time to cross the beam and also with greater difficulty as indicated by a greater number of foot fall from the beam. Instead, treatment with daily PEA-OXA at a dose of 10 mg\kg produced a significant improvement in locomotor ability.

## Conclusions

Then, in conclusion, our results showed how in this sciatic nerve crush model treatment with PEA-OXA at a dose of 10 mg\kg daily for 14 days following the injury was able to reduce the degree of injury the inflammatory process is histologic, also by reducing the number of mast cells, as well as the degeneration of the nerve structure, inhibiting an excessive activation of the apoptotic process and promoting instead the activation of reparative processes. In addition, the treatment with PEA-OXA had a marked analgesic effect, inhibiting the mechanical allodynia and thermal hyperalgesia, then overall, the processes so far described have produced a marked improvement of the locomotor function of the group treated with PEA-OXA compared to the vehicle group.

## Abbreviations

GFAP: Glial fibrillary acidic protein
Iba1: Ionized calcium binding adaptor molecule 1
Ikb-α: Kappa-B inhibitor alpha
IL-1β: Interleukin 1 beta
NAAA: NAE-hydrolyzing acid amidase
NAEs: *N*-Acylethanolamines
Nf-κb: Nuclear factor kappa-B
NGF: Nerve growth factor
PEA: *N-*Palmitoylethanolamide
PEA-OXA: 2-Pentadecyl-2-oxazoline of palmitoylethanolamide
SFI: Sciatic functional index
TNF-α: Tumor necrosis factor alpha
